# The risk factors of polymicrobial periprosthetic joint infection: a single-center retrospective cohort study

**DOI:** 10.1186/s12891-021-04664-0

**Published:** 2021-09-12

**Authors:** Hao Li, Jun Fu, Erlong Niu, Wei Chai, Chi Xu, Li Bo Hao, Jiying Chen

**Affiliations:** 1 Medical School of Chinese PLA, Beijing, People’s Republic of China; 2 Department of Orthopedic Surgery, The First Medical Center, Chinese PLA General Hospital, 28 Fuxing Road, 100853 Beijing, People’s Republic of China

**Keywords:** Periprosthetic joint infection, Total joint arthroplasty, Polymicrobial PJI, Risk factors

## Abstract

**Background:**

Periprosthetic joint infection is a serious complication after total joint arthroplasty and polymicrobial PJI which compose a subtype of PJI often indicate worse outcomes compared to monomicrobial periprosthetic joint infection. However, a literature review suggested that there were limited number studies evaluating the risk factors of polymicrobial PJI.

**Materials and methods:**

Between 2015 January and 2019 December, a total of 64 polymicrobial PJI patients and 158 monomicrobial PJI patients in a tertiary center were included in this study and corresponding medical records were scrutinized. The diagnosis of PJI was based on 2014 MSIS criteria. Logistic regression was used to identify the association between various variables and polymicrobial PJI and ROC curve was used to identify their efficiency.

**Results:**

The prevalence of polymicrobial PJI is 28.3% in our cohorts. After adjusting for the presence of sinus, previous and knee infection, isolation of enterococci (OR, 3.025; 95%CI (1.277,7.164) *p* = 0.012), infection with atypical organisms (OR, 5.032;95%CI: (1.470,17.229) p = 0.01), infection with gram-negative organisms (OR, 2.255; 95%CI (1.011,5.031) *p* = 0.047), isolation of streptococcus spp. (OR, 6; 95%CI (2.094,17.194) *p* = 0.001), and infection with CNS (OfR, 2.183;95%CI (1.148,4.152) *p* = 0.017) were risk factors of polymicrobial PJI compared to monomicrobial PJI. However, knee infection is related to a decreased risk of polymicrobial PJI with an adjusted OR = 0.479 (*p* = 0.023).

**Conclusion:**

This study demonstrated that the prevalence of polymicrobial PJI is 28.3% in PJI patients. Moreover, the presence of sinus tract and previous joint revisions were risk factors for identifying different bacterial species in the intraoperative specimens. Therefore, in these PJI cases, it is necessary to examine multiple specimens of both intraoperative tissue and synovial fluid for increasing the detection rate and obtaining resistance information.

## Backgrounds

Total knee arthroplasty (TKA) and total hip arthroplasty (THA) are successful surgeries during the last century but some severe complications occur after total joint arthroplasty (TJA). Periprosthetic joint infection (PJI) is a serious complication after TJA and lays a huge burden on both medical teams and PJI patients [1–3].

Polymicrobial PJI, accounting for 6 to 37% of PJI, is a subtype of PJI and some studies revealed that polymicrobial PJI was associated with reduced cure rate compared to monomicrobial PJI [2–4]. The management of polymicrobial PJI is tough and requires repeated revisions, higher treatment cost, administration of board-range antibiotics and multiple antibiotics to fight against PJI pathogens. Therefore, the treatment of polymicrobial PJI is associated with higher motility, higher costs, and more complications [5–8].

However, a literature review suggested that the studies about polymicrobial PJI were limited because of the relatively low occurrence rate of polymicrobial PJI. Despite a fact that some studies revealed that the presence of sinus and certain cultured pathogens was associated with a higher risk of polymicrobial PJI, there are still some uncertainties regarding the risk factors of polymicrobial PJI [2, 3, 5]. Moreover, few studies revealed the distribution of polymicrobial PJI pathogens in Asian.

To address the problems mentioned above, we performed a single-center retrospective study to determine 1) the distribution of pathogens in polymicrobial PJI patients 2) the risk factors associated with risk factors of polymicrobial PJI.

## Material and methods

### Patients

**Fig. 1:**
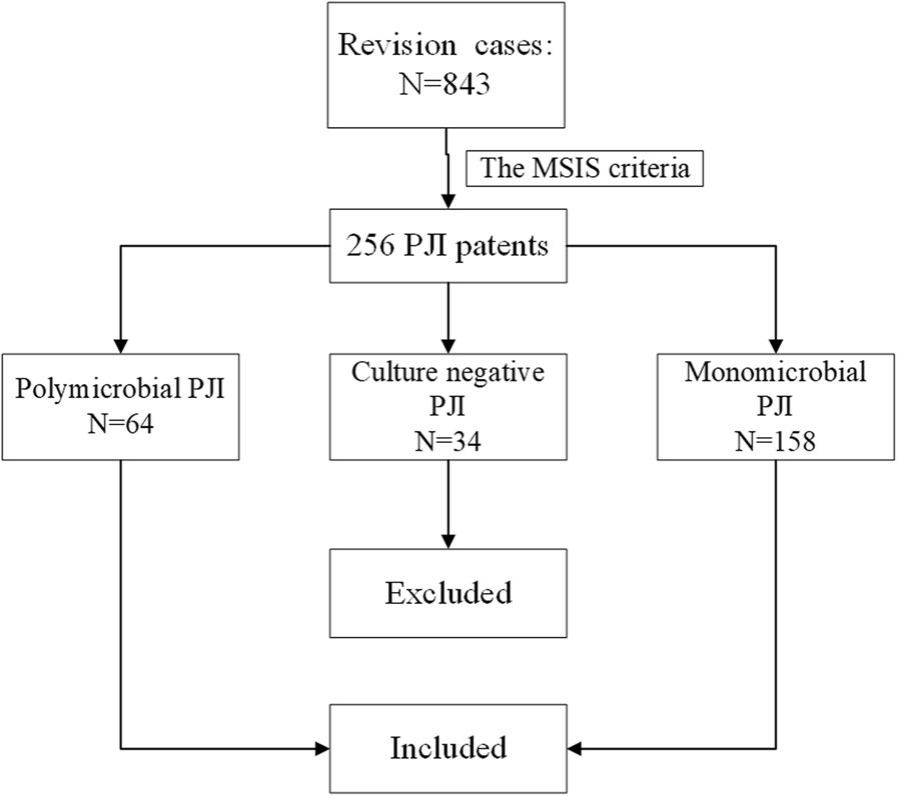
The process of PJI cases included in this study

Institutional review board approval was obtained prior to the commencement of this study and informed consents were obtained before revisions. Between 2015 and 2019, a total of 843 revision patients were included consequentially in this study initially. And the inclusion criteria were as follows: 1) PJI patients based on the 2014 MSIS criteria 2) No spacer implantation. Exclusion criteria: Culture-negative PJI patients were excluded from this study [9]. The corresponding medical records were extracted and then, these data were scrutinized manually. After selection, a total of 256 PJI patients were included in this study and the process of selection was shown in Fig. 1.

Moreover, following information was extracted: the age, sex, body mass index (BMI), involved joint, the type of identified pathogens, previous revisions, the nature of the surgical treatment, the presence of sinus tract, the levels of ESR, CRP. Previous revisions were defined as the patients received surgeries on the same joint after primary total joint arthroplasty.

### The diagnosis of polymicrobial PJI

The PJI diagnosis was based on the 2014 Musculoskeletal Infection Society (MSIS) criteria [9, 10]. The patients with PJI in which at least 2 different organisms were isolated from the culture of synovial fluid or intraoperative tissue were considered to have polymicrobial PJI. A total of 64 PJI patients were considered to have polymicrobial PJI according to this definition.

In our joint center, arthrocentesis was performed routinely before revisions.

### Culture and microbiological analysis

According to the standard protocol at our institution, specimens obtained was sent for aerobic, anaerobic, and fungal cultures, with a mean number of 5 samples taken from each PJI patient. After culture, the pathogens isolated were identified and antibiotic sensitivity tests (AST) were performed. A matrix-assisted laser desorption-isolation time-of-flight mass spectrometer (VITEK-MS (BioM’erieux)) was used to identify the pathogens isolated from culture.

### Statistically analysis

The variables were divided into continuous variables and dichotomous data based on the types of data. A normal distribution test was used to evaluate the distribution of continuous variables. The continuous variables were described as means if the normal distribution was achieved. Otherwise, corresponding medians were calculated. Rand sum test and student t-test were used to detect the difference if the corresponding applicable conditions were met. Dichotomous data were described as frequencies and compared by chi-squared test subsequently. The continuous variable (BMI) was transferred into categorical variables according to the reference standard of Asian: <= 18.5 kg/m^2^, 18.5-24 kg/m^2^, > = 24 kg/m^2^. And the continuous variable (ages) was transferred into binary variables: < 65 years and, > = 65 years. After that, logistic regression was performed. Univariate logistic regressions were performed to identify the potential risk factors associated with polymicrobial PJI within this PJI cohort. Then, a multiple logistic regression analysis was performed to adjust confounding factors (previous revisions and the presence of sinus) and determine the risk factors of polymicrobial PJI within this PJI cohort. OR and adjusted OR were used to evaluate the relative risk of these potential risk factors. The statistical analysis was performed on SPSS (IBM; version 26.0). *P* < 0.05 indicates statistical significance.

## Results

### Demographic characteristic of PJI patients

The mean ages were 59 and 64.2 years in the polymicrobial PJI and monomicrobial groups, respectively and the details of these two groups were summarized in Table 1.

**Table 1:** The demographic characteristics of PJI patients included in this study

characteristic	Polymicrobial	Monomicrobial	*P* value
	PJI *n* = 64	PJI *n* = 158	
Age (years)*	63.5 (38–74)	66 (36–82)	0.012
Knees, n (%)	24 (37.5%)	94 (59.5%)	0.003
Male, n (%)	27 (42.2%)	70, (44.9%)	0.773
Body Mass Index*(Kg/m2)	25.6 (18.4–33.3)	25.3 (15.6–48.6)	0.637
Sinus tract, n (%)	31 (48.4%)	17 (10.8%)	< 0.001
CRP * (mg/dL)	1.26(0.3–5.6)	3.37(0.07–20.9)	0.385
ESR * (mm/h)	44(8–123)	51 (4–111)	0.489
Previous revisions, n (%)	30 (46.9%)	43(27.2%)	0.005
PJI	25(83.3%)	31(72.1%)	
Aseptic loosening	4(13.33%)	2(4.6%)	
Dislocation	0	1(2.3%)	
Periprosthetic fracture	0	2(4.6%)	
Other reasons	1(3.33%)	7(16.3%)	
Acute PJI**	9(14.1%)	20(12.7%)	0.779
Synovial fluid polymorphonuclear neutrophils (%) *	63(70–96)	64(3–98)	0.923
Synovial fluid white blood-cell count (cells/mL) *	10,600(260–66,400)	15,440(300–102,580)	0.907

* Values were given as medians with the range in the parentheses

** Acute PJI: the PJI occurred with first postoperative month

### The distribution of pathogens in PJI patients

The percentage of *S. aureus* in polymicrobial PJI and monomicrobial PJI was 11.9 and 18.4%, respectively. The percentage of CNS in the two group was 32.1 and 35.4%, respectively. The percentage of fungus in the two group was 0 and 7.6%, respectively. The percentage of gram-positive bacillus in the two group was 6.4 and 5.1%, respectively. The details about the distribution of pathogens in PJI patients was shown in Table 2.

**Table 2:** The distribution of pathogens in monomicrobial PJI and polymicrobial PJI

Pathogens	Polymicobial PJI n, (%)	Monomicrobial PJI n, (%)	*P* value
*S. aureus*	13, (11.9%)	29, (18.4%)	0.156
CNS	35, (32.1%)	56, (35.4%)	0.572
Streptococcus spp.	13, (11.9%)	7, (4.4%)	0.022
Enterococcus spp.	14, (12.8%)	15, (9.5%)	0.387
Gram negative	16, (14.7%)	18, (11.4%)	0.428
Fungus	0, (0%)	12, (7.6%)	0.002
*E.coli*	3, (2.7%)	8, (5.1%)	0.533
Gram-positive bacillus	7, (6.4%)	8, (5.1%)	0.636
Atypical pathogens	8, (7.3%)	5, (3.2%)	0.205
Total	109	158	

### The risk factors of polymicrobial PJI

In the multivariate logistic regression analysis after adjusting BMI and ages, some characteristics were associated with polymicrobial PJI: the presence of a sinus tract (OR, 2.959 [95% CI, 1.565 to 5.596]; *p* = 0.001), the presence of previous revisions (OR, 1.954 [95% CI, 1.036 to 3.686]; *p* = 0.039). In contrast, knee infection (OR, 0.479 [95% CI, 0.255 to 0.903]; *p* = 0.023) is associated with decreased risk of polymicrobial PJI. Besides, in the multivariate logistic regression analysis, the adjusted OR of different pathogens was calculated after adjusting the presence of sinus, previous revision, and knee infection. After adjusting these factors, isolation of enterococci (OR, 3.025 [95% CI, 1.277 to 7.164]; *p* = 0.012), infection with atypical organisms (OR, 5.032 [95% CI, 1.470 to 17.229]; *p* = 0.01), infection with gram-negative organisms (OR, 2.255 [95% CI, 1.011 to 5.031]; *p* = 0.047), isolation of streptococcus spp. (OR, 6 [95% CI, 2.094 to 17.194]; p = 0.001), and infection with CNS (OR, 2.183 [95% CI, 1.148 to 4.152]; *p* = 0.017) are the risk factors of polymicrobial PJI. The details about the risk factors of polymicrobial PJI were shown in Table 3.

**Table 3:** The risk factors associated with polymicrobial periprosthetic joint infection

Factors	OR	Adjusted OR	*P*-Value
***Host factors***			
**BMI**			
BMI (< 18.5 kg/m2)	0.947(0.505,1.777)	0.338(0.037,3.067)	0.335
BMI (> 24 kg/m2)	0.329(0.039,2.759)	1.445(0.722,2.894)	0.298
Sinus *	3.405(1.832,6.330)	2.959(1.565,5.596)	0.001*
Age (> 65 years) *	0.483(0.268,0.873)	0.554(0.298,1.032)	0.063
Previous revisions*	2.512(1.375,4.589)	1.954(1.036,3.686)	0.039*
Knee*	0.402(0.221,0.731)	0.479(0.255,0.903)	0.023*
***Pathogens***			
***S. aureus***	1.134(0.546,2.353)	0.624(0.268,1.451)	0.273
**CNS**	1.773(0.987,3.184)	2.183 (1.148, 4.152)	0.017*
**Streptococcus spp.**	5.499(2.080,14.536)	6 (2.094,17.194)	0.001*
**Enterococcus spp.**	2.669(1.204,5.919)	3.025(1.277,7.164)	0.012*
**Gram negative**	2.593(1.226,5.483)	2.255(1.011,5.031)	0.047*
***E. coli***	0.922(0.237,3.592)	0.840(0.198,3.570)	0.814
**Fungus**	< 0.001(< 0.001, ∞)	< 0.001(< 0.001, ∞)	0.999
**Atypical pathogens**	4.371(1.372,13.924)	5.032(1.470,17.229)	0.01*

*CNS* coagulation negative staphylococcus

*P < 0.05

**The values were given as means with the 95%CI in the parentheses

* The values were given as the number of pathogens with the percentage in the parentheses

### The number of pathogens detected by synovial fluid culture and tissue cultures

**Fig. 2:**
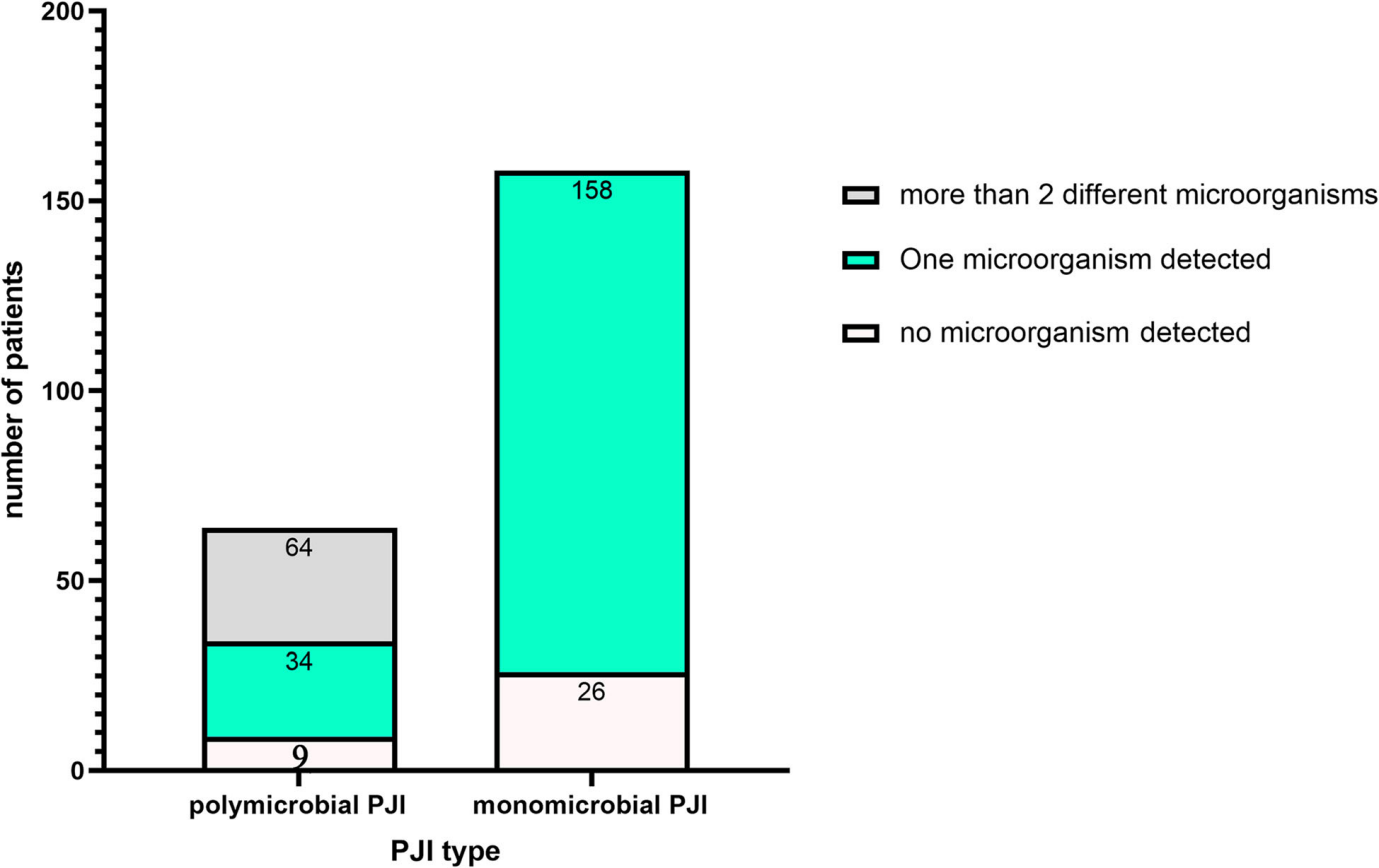
The number of Photogens detected by Preoperative Synovial Fluid Cultures

In our joint center, arthrocentesis was performed routinely before revisions, in 46.9% of polymicrobial PJI (30/64), at least two different microorganisms were isolated in the preoperative synovial fluid cultures. In 39.1% of polymicrobial PJI (25/64), only one microorganism was detected, and the additional microorganisms were detected in the intraoperative tissue cultures. The details were shown in Fig. 2.

## Discussion

Polymicrobial PJI is a serious complication after total joint arthroplasty and often indicates unfavorable prognosis compared to monomicrobial PJI. However, the studies about the risk factors of polymicrobial PJI are limited. This study evaluated the risk factors of polymicrobial PJI and revealed some findings.

Culture-negative PJI weren’t included from this study because previous antibiotics administration is a major cause of culture-negative PJI. Polymicrobial PJIs accounted for 10.5 to 19% of THA PJIs and 9 to 12.3% of TKA PJIs in previous studies [3]. We have observed that the percentage of polymicrobial PJI in culture-positive PJI is about 28.8% and it is consistent with previous reports [11].

The distribution of pathogens in polymicrobial PJI is like that in monomicrobial PJI patients. Staphylococcus spp., including *staphylococcus aureus* and CNS, is still the most comm isolated pathogen in polymicrobial PJI. However, the percentage of streptococcus spp. in polymicrobial PJI is relatively higher than that in monomicrobial PJI. Our results were consistent with previous studies.

The presence of sinus tract is associated with higher risk of polymicrobial PJI and this result is consistent with previous studies [2, 3]. The sinus tract can become a shelter of pathogens and a pathway for causative agents to enter the joint cavity so that polymicrobial infection is more common in PJI patients with sinus tracts. Therefore, surgeons can obtain more specimens for cultures during revisions in PJI patients with sinus tracts with a bid to improve the detection rate of PJI pathogens by which antibiotic can be administrated more precisely.

Some studies suggested that repeated revision was a risk factor of PJI but the association between previous revisions wasn’t revealed by other studies [1, 2]. Here, our studies revealed that the presence of previous revisions was also a risk factor of polymicrobial periprosthetic joint infection. Previous repeated procedures in the same joint can impair the surrounding blood supplies around the joints potentially and then, the joints became vulnerable to various pathogens because of the poor status of the soft tissues in these patients. It can be the reason of higher risk of polymicrobial PJI in PJI patients with repeated revisions. Hence, polymicrobial PJI should be specially noted in these PJI patients and board-range antibiotics and more specimens for cultures can be optional.

Interestingly, Cantey et. reported that age 65 years or older was associated with polymicrobial PJIs [2]. However, our result suggested that the mean and median ages in polymicrobial PJI cohort were lower than those in monomicrobial PJI cohort. And the OR of age 65 years or older is 0.483 in the bivariate logistic regression ([95% CI, 0.268 to 0.873], *P* value = 0.018). To further clarify the association between age and polymicrobial PJI, two confounding variables (the presence of sinus and previous revisions) was adjusted, and the OR after adjusting was 0.554 ([95% CI, 0.298 to 1.032]; *p* = 0.063). This result suggested that there are potential association between previous revisions and the younger ages. Considering that younger patients require a higher quality of life and they are more aggressive in the choice of surgery than the elder ones, the negative contribution of age to polymicrobial PJIs may be attributed to previous revisions.

To further analyze the relationship between previous revisions and polymicrobial PJI, the reasons of previous revisions were collected and analyzed. Previous PJI is the major cause of previous revisions (56 cases), followed by aseptic loosening (6 cases). Then, bivariate logistic regression was performed to analysis the relationship between the two factors and polymicrobial PJI. Our result revealed that they were risk factors of polymicrobial PJI. However, it is hard to discern whether the revision is the cause or the reason of polymicrobial PJI because several PJI patients received revisions in other joint center where the protocols of pathogens culture is heterogeneous and previous revisions can also be the consequence of polymicrobial PI where polymicrobial PJI are associated with increased number of revisions operations and lower cure rate compared to monomicrobial PJI [8]. However, polymicrobial PJI should be noted when a PJI patient with previous revisions was admitted and then, optimal pathogen detection protocols can be chosen such as repeated joint aspiration and more periprosthetic tissues for culture when revisions are performed [12–14].

In line with prior studies, certain types of pathogens are more likely to be isolated from polymicrobial periprosthetic joint infection compared to monomicrobial PJI such enterococcus, streptococcus, CNS and atypical pathogens [2–5, 15, 16]. Doctors should take this fact into account to guide antibiotics administration when these pathogens were isolated from PJI patients. Besides, the interesting association between certain pathogens and polymicrobial PJI patients indicated that polymicrobial periprosthetic joint infection can be noted when these pathogens were cultured from PJI patients and board-range antibiotics can be optional in PJI patients with these pathogens. Moreover, we also performed a correlation analysis to explore the positive association between different pathogens in polymicrobial PJI group. Unfortunately, no positive association was detected (not shown in this study). It can be attributed to the relatively small sample size in the PJI group. Therefore, further multi-center studies focusing on the association between the pathogens in polymicrobial PJI is necessary because the clarification on this association can guide empirical antibiotic use more precisely. And molecule diagnostics with high sensitivity such as the metagenomic next-generation sequencing (mNGS) can be used to detect polymicrobial PJI when these pathogens were detected by cultures [17].

There were several limitations in this study. Firstly, this study was performed in a tertiary joint center retrospectively and thus has some inherent limitations and selection bias. For example, previous antibiotics can affect microbiological results but the history of antibiotics administration can’t be evaluated comprehensively in this study because of the limited medical records. Therefore, we exclude culture-negative PJI patients from this study in a bid to lower this effect. However, bias was also added after exclusion. Secondly, the prognosis of these PJI patients was not included in this study and this field needs to be explored further. Thirdly, only knees and hips were included in this study and no other joints were evaluated in this study. This nature of the study design can also add some bias to this study and further studies including other joints are necessary. Finally, no co-infective bacterial and fungal PJIs were included in this study but Stevenson et. reported that these patients suffered from unfortunate outcomes. Therefore, the risk factors of co-infective bacterial and fungal PJIs need to be explored further [18].

## Conclusions

The studies about polymicrobial PJI was limited. Here, our study reveals some risk factors of polymicrobial periprosthetic joint indention. In line with previous studies, polymicrobial PJI was associated with the presence of sinus tract. Besides, the presence of previous revisions was also risk factors of polymicrobial PJI, whereas knee infection is associated with a decreased risk of polymicrobial PJI. Moreover, certain types of pathogens such as CNS, enterococcus, streptococcus spp. and atypical pathogens were more common in the polymicrobial PJI than those in the monomicrobial PJI patients. These risk factors should be considered when antibiotics administration was performed.

## Data Availability

All data and materials were in full compliance with the journal’s policy. And the data were obtained in Department of Orthopedic Surgery, The First Medical Center, Chinese PLA General Hospital. The datasets used and during the current study are available from the corresponding author on reasonable request.

## Abbreviations

TJA: Total joint arthroplasty
PJI: periprosthetic joint infection
MSIS: Musculoskeletal Infection Society criteria
CNS: coagulase negative staphylococci
